# NetBCE: An Interpretable Deep Neural Network for Accurate Prediction of Linear B-cell Epitopes

**DOI:** 10.1016/j.gpb.2022.11.009

**Published:** 2022-12-13

**Authors:** Haodong Xu, Zhongming Zhao

**Affiliations:** 1 Center for Precision Health, School of Biomedical Informatics, The University of Texas Health Science Center at Houston, Houston, TX 77030, USA; 2 Human Genetics Center, School of Public Health, The University of Texas Health Science Center at Houston, Houston, TX 77030, USA; 3 The University of Texas MD Anderson Cancer Center UTHealth Houston Graduate School of Biomedical Sciences, Houston, TX 77030, USA; 4 Department of Biomedical Informatics, Vanderbilt University Medical Center, Nashville, TN 37203, USA

**Keywords:** B-cell epitope, Immunotherapy, Deep learning, Machine learning, Vaccine development

## Abstract

Identification of **B-cell epitopes** (BCEs) plays an essential role in the development of peptide vaccines and immuno-diagnostic reagents, as well as antibody design and production. In this work, we generated a large benchmark dataset comprising 124,879 experimentally supported linear epitope-containing regions in 3567 protein clusters from over 1.3 million B cell assays. Analysis of this curated dataset showed large pathogen diversity covering 176 different families. The accuracy in linear BCE prediction was found to strongly vary with different features, while all sequence-derived and structural features were informative. To search more efficient and interpretive feature representations, a ten-layer **deep learning** framework for linear BCE prediction, namely NetBCE, was developed. NetBCE achieved high accuracy and robust performance with the average area under the curve (AUC) value of 0.8455 in five-fold cross-validation through automatically learning the informative classification features. NetBCE substantially outperformed the conventional **machine learning** algorithms and other tools, with more than 22.06% improvement of AUC value compared to other tools using an independent dataset. Through investigating the output of important network modules in NetBCE, epitopes and non-epitopes tended to be presented in distinct regions with efficient feature representation along the network layer hierarchy. The NetBCE is freely available at https://github.com/bsml320/NetBCE.

## Introduction

B-cell epitopes (BCEs) represent the regions on antigen surfaces where designated antibodies recognize, bind to, and subsequently induce the immune response in humoral immunity [1], [2]. Identification of BCEs is a crucial step in immunological studies and medical applications, including peptide-based vaccine development, antibody production, and disease prevention [3]. BCEs are commonly classified into two types: linear epitopes and conformational epitopes. Linear epitopes are composed of a linear sequence of residues from an antigenic sequence, while conformational epitopes refer to atoms on surface residues that come together via protein folding [4]. Many experimental approaches have been developed for BCE identification, including peptide microarrays, X-ray crystallography, and enzyme-linked immunosorbent assay (ELISA) [5]. However, these approaches are expensive and resource intensive. On the other hand, computational approaches have demonstrated promise for predicting linear BCEs. So far, many computational approaches have been published for linear BCE prediction from proteins’ primary sequences or antigens’ 3D structures (Table S1) [6].

These initially developed methods such as Antigenic [7], PREDITOP [8], PEOPLE [9], BEPITOPE [10], and BcePred [11] typically used and characterized single or a subset of amino acid physicochemical properties, such as hydrophobicity [12], surface accessibility, flexibility [13], and antigenicity [14]. Recently, due to the booming of generation of BCE data, the next-generation approaches have attempted to apply some machine learning (ML) algorithms for BCE prediction. One of the most representative and reliable methods was BepiPred-1.0 [15], combining a hidden Markov model (HMM) with an amino acid propensity scale. Moreover, other ML algorithms were adopted in tools developed afterward, including the Naïve Bayes algorithm in Epitopia [16], neural networks in ABCpred [17] and GFSMLP [18], and support vector machine (SVM) in the vast majority of predictors including BCPred [19], COBEpro [20], AAPPred [20], SVMTriP [21], BEEPro [22], LBtope [23], LBEEP [24], APCpred [25], and BCEPS [26]. The differences of these methods include the dataset construction, feature encoding and selection, and the hyperparameter optimization of the SVM, among others. More feature encoding strategies based on sequence-derived and structural information were utilized, including amino acid composition (AAC), BLOSUM62 scoring matrix, accessible surface area (ASA), secondary structure (SS), and backbone torsion angles (BTAs) [27], [28]. Using the multiple linear regression, a new method, named EPMLR [29], was developed for epitope classification. Additionally, different types of deep neural network (DNN) have also been implemented in the task of BCE prediction, such as deep maxout networks in DMN-LBE, deep ridge neural network in DRREP [30], and bidirectional long short-term memory (BLSTM) in a recent method named EpiDope [31]. In 2017, BepiPred-2.0 [15] was released, which was trained only on crystal structure information using a random forest (RF) algorithm. Ensemble learning framework combining multifeature and model was also used in methods such as iBCE-EL [32] and iLBE [33]. However, which features are the most informative for BCE prediction remains unclear. Most of these methods have been developed using conventional ML algorithms, which may be less powerful in feature representation than deep learning algorithms [34], [35], [36], [37]. Recently, several hundred thousand high-quality linear BCE assay datasets have been stored in the Immune Epitope Database (IEDB) [38]. This large collection provides a unique opportunity to further develop computational approaches for identification of potential linear BCEs from protein sequences.

In this work, we first collected and curated over 1.3 million B cell assays from the IEDB database. Through quality control procedures, we compiled an experimentally well-characterized dataset, containing more than 124,000 experimentally linear epitope-containing regions from 3567 protein clusters. The curated dataset covered 176 different families, indicating strong pathogen diversity. After homology clearance, we carefully evaluated five types of sequence-derived features [39], six clusters of physicochemical properties [40], [41], as well as three types of structural features [42] using six conventional ML algorithms on the curated dataset. The results show that different types of features displayed various accuracies for linear BCE prediction and all features were informative. With a sufficient training dataset of B cell assays, the deep neural network can automatically learn informative classification features, making it very appropriate for linear BCE prediction [43]. Therefore, we developed NetBCE, a ten-layer deep learning framework, and implemented it into tool. The epitope sequences were encoded and taken as input for subsequent feature extraction and representation in the convolution–pooling module. A BLSTM layer was added to retaining features over a long duration and to facilitate the model catching the combinations or dependencies among residues at different positions. Lastly, an attention layer was joined to link the BLSTM layer and the output layer. NetBCE outperformed conventional ML methods by an improvement of the area under curve (AUC) value in a range of 8.77%–21.58% using the same training dataset. Moreover, in our comparison of NetBCE with other existing tools using an independent dataset, NetBCE achieved performance with the AUC value of 0.8400, and had AUC value improvement by over 22.06% for the linear BCE prediction when compared to other tools. To elucidate the capability of hierarchical representation by NetBCE, we visualized the epitopes and non-epitopes using Uniform Manifold Approximation and Projection (UMAP) [44] based on the feature representation at various network layers. We found that feature representation became more discriminative further along the network layer hierarchy. More specifically, the feature representations for epitope and non-epitope sites were mixed at the input layer. As the model continued to train, epitopes and non-epitopes tended to be presented in distinct regions with efficient feature representation. The NetBCE tool, which is available at https://github.com/bsml320/NetBCE, allows the user to explore the data and prediction results in an easily readable and interpretable manner.

## Method

### Data collection and processing

**Figure 1:**
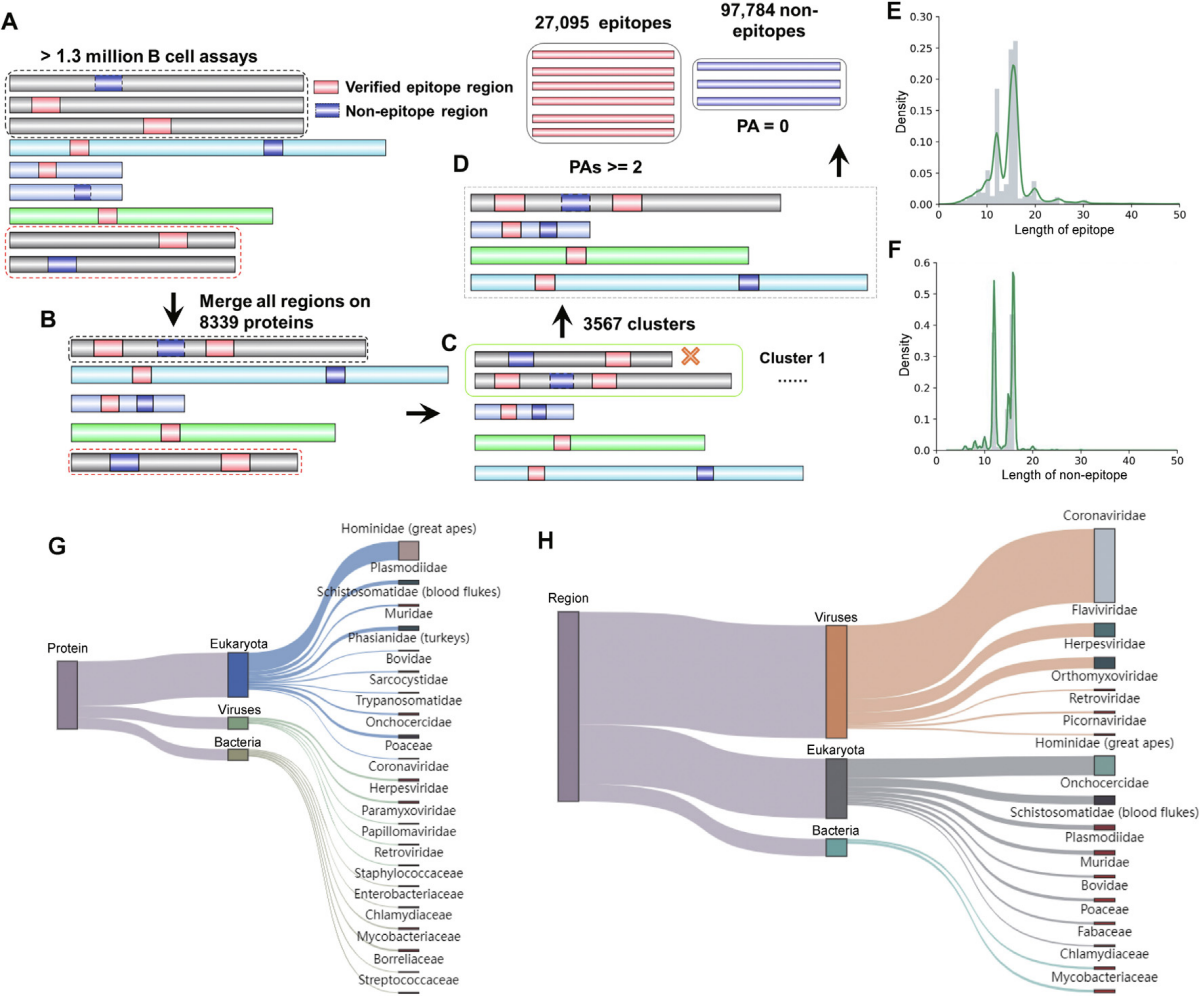
**Benchmark data preparation and evaluation** **A.** The experimentally identified epitope-containing regions were collected from the IEDB database. **B.** Identical protein sequences were integrated and the verified epitope regions were aggregated. **C.** Sequence redundancy was cleaned for the similar proteins by CD-HIT. **D.** Proteins with the largest number of epitope-containing regions were retained. The curated dataset was divided into epitopes and non-epitopes according to epitope assay information. We defined all epitope-containing regions that were tested by at least two PAs as epitopes to avoid possible chance of a single test result. Moreover, all epitope-containing regions that were tested in at least two assays but not tested as positive in any assay were stored as non-epitopes. All other epitope-containing regions with inconsistent test responses that did not meet both criteria were excluded. **E.** The length distribution of epitopes. **F.** The length distribution of non-epitopes. **G.** Taxonomic distribution in super-kingdoms and families at the protein level. **H.** Taxonomic distribution in super-kingdoms and families at the verified epitope level. PA, positive assay.

To establish a reliable model, an experimentally supported dataset was compiled as follows (Figure 1). First, we downloaded over 1.3 million B cell assays from the IEDB (https://www.iedb.org/), the most comprehensive database holding the largest number of experimentally identified epitopes and non-epitopes. Each entry contained an antigen protein sequence with a marked region (hereafter termed “epitope-containing region”) that was an experimentally verified epitope or non-epitope. Protein sequences were retrieved from the National Center for Biotechnology Information (NCBI) [45] and the Universal Protein Resource (UniProt) database [46] based on the antigen protein IDs provided in the epitope entry. We preprocessed and filtered the dataset by several criteria (Figure 1). First, identical protein sequences were integrated, and all related information about epitope-containing regions was aggregated. Second, sequence redundancy for those proteins of non-identical but highly similar was cleared. Using CD-HIT program [47], all proteins were clustered with a threshold of 90% sequence similarity. For each cluster, only the protein having the largest number of epitope-containing regions was retained. To ensure high confidence of the dataset, each epitope assay was carefully explored and regarded as a positive hit only when it has been tested as positive in two or more different B cell assays, whereas those regions that were tested in at least two assays but all were not positive were considered as non-epitopes. In addition, we excluded 1900 candidate epitopes that had less than 5 or more than 25 amino acid residues from the dataset (changed 126,779 to 124,879). The number of such epitopes accounted for only a small portion (approximately 1%), but an inclusion of them may result in outliers during model development. Overall, the final non-redundant dataset for training and testing contained 27,095 positive and 97,784 negative epitope-containing regions from 3567 protein sequence clusters, respectively. The compiled dataset was divided into the training dataset (90% of the total epitope-containing regions) (Table S2) and the independent dataset (10% of the remaining epitope-containing regions) (Table S3).

### Feature encoding

One main goal is to benchmark the ability of various feature encoding strategies implemented in previous tools to correctly predict linear BCEs. Based on our curated benchmark dataset, 14 types of features were encoded from the epitope-containing regions of both the positive and negative datasets. These datasets included five types of sequence-derived features, six clusters of physicochemical properties, and three structural features. We classified these 14 feature types as follows. 1) AAC, which counts the frequencies of 20 types of typical amino acids in epitope-containing regions. 2) Binary, which denotes position-specific composition of the amino acids. The 20 types of amino acids were alphabetically sorted and each amino acid was transformed into a binary vector. 3) Composition of K-spaced amino acid pairs (CKSAAP), which calculates the composition of amino acid pairs that are separated by *k* other residues within epitope-containing regions. 4) Physicochemical properties, which represent amino acid indices of various physicochemical properties. Numerous studies have indicated strong correlations between physicochemical properties of amino acids and BCEs. In this study, we employed and encoded six categories of properties. They are α and turn propensities (AAindexClusterA), β propensity (AAindexClusterB), AAC features (AAindexClusterC), hydrophobicity (AAindexClusterH), physicochemical properties (AAindexClusterP), and other properties that do not belong to the aforementioned five clusters (AAindexClusterO). 5) Enhanced AAC (EAAC), which represents the local AAC for the fixed-length sequence window that continuously slides from the 5′ to 3′ terminus of each protein sequence. 6) BLOSUM62 scoring matrix, which is commonly used to score the alignments between evolutionarily divergent protein sequences. 7) ASA, which indicates the exposed area of an amino acid residue to solvent. The SPIDER2 tool [42] computes a potential ASA value for each amino acid in epitope-containing regions. 8) SS, which represents three types of structural elements, including α-helix, β-strand, and coil. 9) BTA, which measures continuous angle information of the local conformation of proteins, including the BTAs *φ* and *Ψ*, the angle between Cα_i-1_-Cα_i_-Cα_i+1_ (*θ*), and the dihedral angle rotated about the Cα_i_-Cα_i+1_ bond (*τ*). More detailed feature description and classification are summarized in Table S4.

### NetBCE model construction

**Figure 2:**
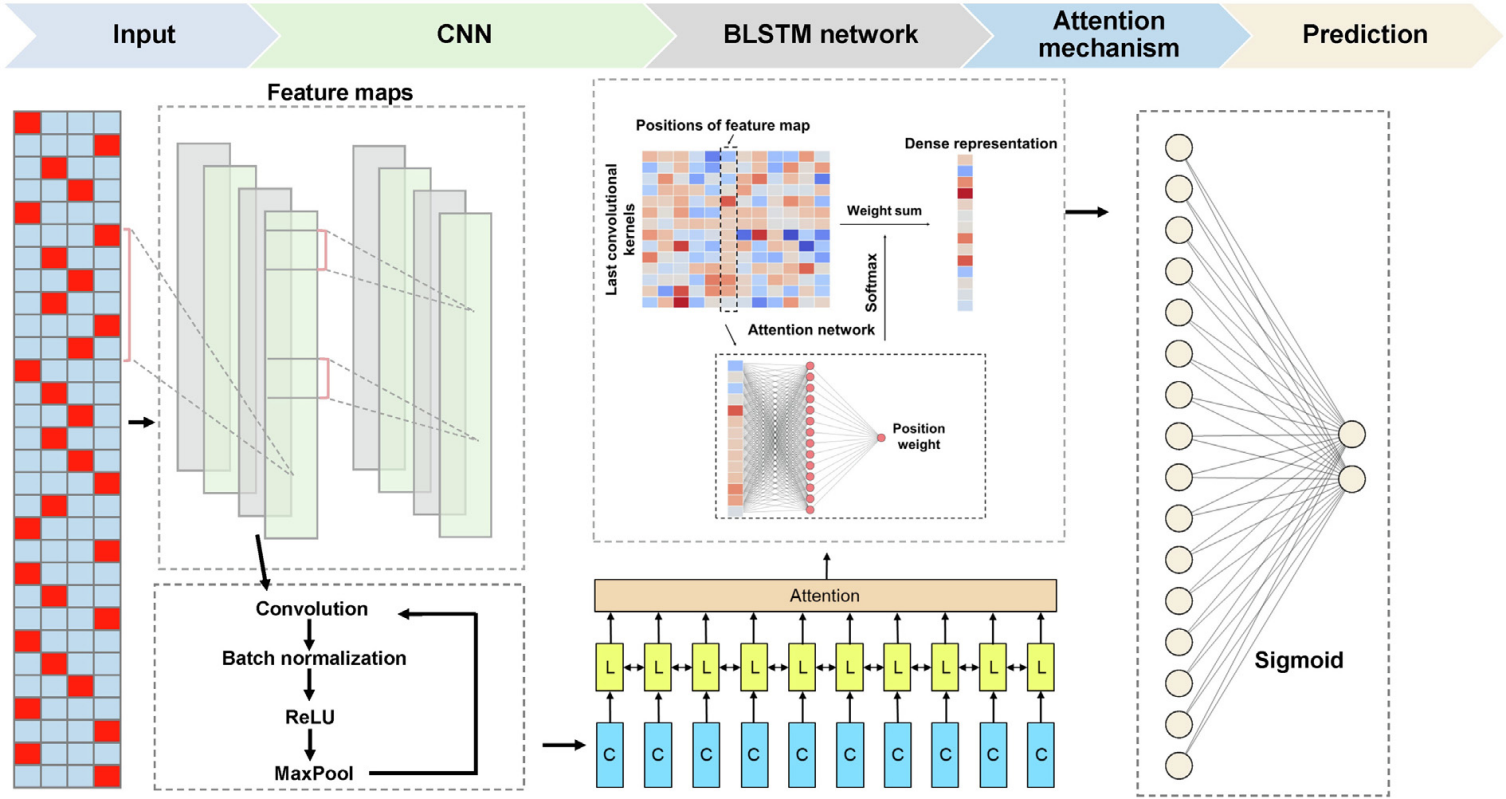
**Deep learning framework of NetBCE** NetBCE is built on a ten-layer deep learning framework. The epitope sequences were encoded as binary matrix and taken as input. Then, CNN module was used for feature extraction and representation. The activation function is the ReLU being applied to the convolution results, where positive values remain unchanged and any negative values are set 0. BLSTM layer was added for retaining features from a long duration to capture the combinations or dependencies among residues at different positions. A fully connected layer was used to integrate the variables’ output from the attention layer and learn the nonlinear relationship. The output layer was composed of one sigmoid neuron for calculating a prediction score for a given peptide. The sigmoid function is also referred to a squashing function, because its domain is defined as the set of all real numbers, and its range is (0, 1). CNN, convolutional neural network; ReLU, rectified linear unit; BLSTM, bidirectional long short-term memory; L, layer; C, convolution.

**Supplementary Figure S1:**
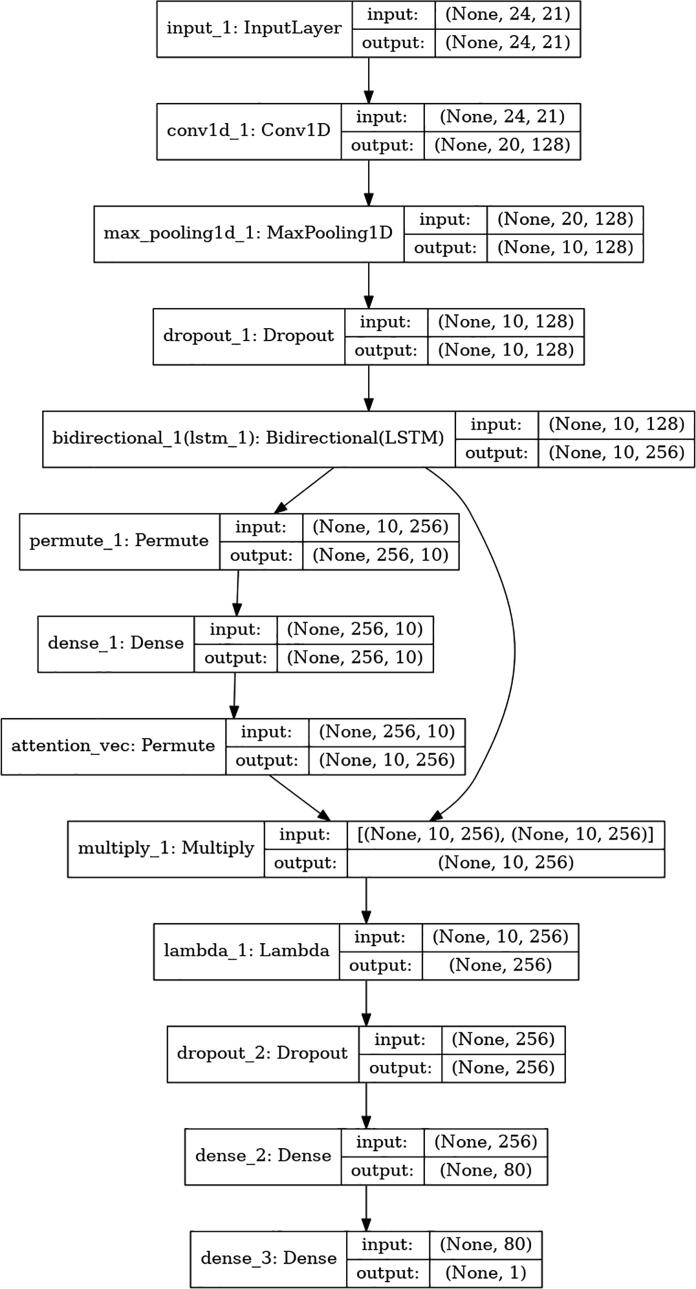
**Schematic diagram of the overall structure of NetBCE**

As shown in Figure 2 and Figure S1, a ten-layer deep learning framework, named NetBCE, was implemented to predict BCEs using amino acid sequences as input. Each layer contained a number of computational units called neurons, which constitutes an internal feature representation. We applied one-hot encoding to convert the epitope sequences to a *L* × 20 binary matrix, where *L* represents the length of the epitope sequence. Then, the binary matrix was entered to a convolution layer [48] to catch sequence sub-motifs. Convolutional kernels act as the crucial components of the convolution layer, which was widely used for sequence motif recognition, regardless of their position in the sequence. A number of studies have used kernels in the convolutional layer to catch sequence patterns from massive sequence data. In the NetBCE, representative patterns were first detected by numerous convolution kernels from the input epitope sequences. The convolutional layer was followed by a maxpooling layer to calculate the maximum activation spots over spatially adjacent regions, and then to summarize the most activated pattern in the sequences. Down sampling strategy in maxpooling downsizes the feature dimension and thus strengthens the deep learning model robustness. To further extract the extensive dependencies of long-range sequence among detected patterns from both forward and backward directions, we added a BLSTM layer [49] in NetBCE. The rationale for adding a BLSTM is that the binding between BCE and B cell receptor (BCR) may be regulated by multiple spaced amino acids. The power of BLSTM for retaining features from a long duration facilitates the model to capture the combinations or dependencies among residues at different positions. The unit in BLSTM contains four parts: three gates (input, forget, and output) and a single cell remembering features over arbitrary intervals. Specifically, considering an epitope sequence with length *L* as input ${\left\{x_{p}\right\}}_{p=1}^{L}$ in BLSTM, and for every position *p*, denote the input gate as *I_p_*, forget gate as *F_p_*, output gate as *O_p_*, hidden state as *H_p_*, and cell state as *C_p_*. The process of BLSTM training is as follows:

(1) $$F_{p}=\sigma \left(W_{f}\times \left[x_{p},H_{p}-1\right]+b_{p}\right)$$

(2) $$I_{p}=\sigma \left(W_{I}\times \left[x_{p},h_{p}-1\right]+b_{I}\right)$$

(3) $$C_{p}=F_{p}\times C_{p-1}-I_{P}\times tanh\left(W_{C}\times \left[x_{p},h_{p}-1\right]+b_{C}\right)$$

(4) $$O_{p}=\sigma \left(W_{O}\times \left[x_{p},h_{p}-1\right]+b_{O}\right)$$

(5) $$H_{p}=O_{p}\times tanh\left(C_{p}\right)$$

To further recognize the most representative sequence patterns in NetBCE, an attention layer [50] was added following the BLSTM layer. Because the most distinct patterns may be located somewhere of the epitope, the attention layer was thus adopted to find more informative features by learning the whole hidden states of the BLSTM layer and distribute higher weights to the important locus. Mathematically, by obtaining the hidden variables ${\left\{B_{p}\right\}}_{p=1}^{L}$ from BLSTM layer as inputs, the attention layer returns the output vector *A* as shown below:

(6) $$\alpha_{p}=\frac{exp(w\left(B_{p}\right))}{\sum_{i=1}^{L}exp\left(w\left(B_{i}\right)\right)}$$

(7) $$A=\sum_{p=1}^{L}\alpha_{p}B_{p}$$

where *w* represents a fully connected neural network that computes a scalar weight.

Finally, we utilized a fully connected layer to integrate the variables output from the attention layer and learn the nonlinear relationships. The output layer was composed of one sigmoid neuron calculating a *S_BCE_* score for a given peptide *y*, as defined as:

(8) $$S_{\mathrm{BCE}}\left(y\right)=sigmoid\left(y\right)=\frac{1}{1+e^{-y}}$$

The *S_BCE_* value, ranging from 0 to 1, represents the probability of peptide to be a real BCE.

### Model training and evaluation

We trained the NetBCE using the Adam optimizer with mini-batch algorithm. The deep learning model was trained to minimize the loss of binary cross-entropy, which catches the difference between the target and predicted label. After each epoch of training, the model was evaluated on the validation dataset, and the corresponding loss and accuracy values were recorded. We introduced an early stop mechanism during training to avoid model overfitting. Specifically, the model was constantly learned until the validation accuracy stopped to increase for twenty epochs. After model training was completed, we evaluated the performance using a test dataset and several metrics were calculated, including accuracy (Acc), sensitivity (Sn), specificity (Sp), and the area under the receiver operating characteristic (ROC) curve (AUC).

The hyperparameters of NetBCE model were optimized to achieve optimal performance using Hyperopt tool [51] via Bayesian mechanism from a list of multiple parameters, including the number of convolutional filters, kernel size, the learning rate, degree of momentum, mini-batch size, strength of parameter regularization, and dropout probability. Hyperopt optimizes the hyperparameter space by creating a classification model upon the metric of the objective function. The probability model was updated after each evaluation of the objective function by incorporating new results. Specifically, 100 evaluations were executed using separate training (inner loop) and validation sets (outer loop). The performance of each set of parameters was evaluated and the corresponding AUC values were calculated. We selected the group of parameters with the highest AUC values as the final parameters of the model. NVIDIA Tensor Cores with four Tesla V100 were used. The Keras version 2.3, a highly useful neural network Application Programming Interface (API), and the TensorFlow-GPU 1.15 version were adopted for a rapid parallel computing.

### Conventional ML classifiers

In this study, we implemented 84 classical ML models for prediction of BCEs based on 14 features using six algorithms: AdaBoost (AB), decision trees (DT), Stochastic Gradient Descent (SGD), k-nearest neighbors (KNN), logistic regression (LR), and RF. Five-fold cross-validation (CV) was performed for each classifier to evaluate the predictive capacity. The ROC curves were illustrated for Sn *vs.* 1 − Sp scores and the AUC values were subsequently calculated. For accurate estimation of the performance, the five-fold CV was independently performed by 10 times and the average AUC value was calculated for each model setting. To determine the best parameters for each model, we tested dozens or hundreds of different parameter combinations for each model, and selected the optimal parameters through multiple CV evaluations.

## Results

### The curated dataset contains large pathogen diversity

From the IEDB database, we extracted over 1.3 million B cell assays with experimentally verified epitope-containing information (Figure 1A). After merging all the identical protein sequences, we obtained 8339 proteins preserving 213,700 verified epitope-containing regions (Figure 1B). After removing the redundancy by CD-HIT software, 3567 protein sequence clusters were identified. This procedure reduced the number of epitope-containing regions by 40.67% (from 213,700 to 126,779; Figure 1C). By applying our quality control procedures, the final filtered dataset contained 3567 proteins with 27,095 epitopes and 97,784 non-epitopes for model construction (Figure 1D). More specifically, the subset of epitopes had an average length of 15.45 amino acids, while the subset of non-epitopes had an average length of 13.97 amino acids. Among all the epitopes, the peptides with lengths of 16, 15, and 12 amino acids accounted for the largest proportion, *i.e.*, 24.99%, 23.72%, and 17.72%, respectively (Figure 1E and F). We then analyzed the taxonomic origin of the protein sequences, as provided by the filtered dataset, and visualized the distribution of species (Figure 1G and H). At the protein level, the curated dataset contained 176 different families. The 21 families with the largest number of epitopes are shown in Figure 1G. The numbers of epitopes in Bacteria, Eukaryota, and Viruses accounted for 16.65%, 65.59%, and 17.76% of all the proteins, respectively, in the curated dataset. At the epitope-containing region level, the proportions showed differently from the protein level. For example, the proportion of Viruses was 59.41%, higher than those of Bacteria (9.17%) and Eukaryota (31.42%). Overall, the curated dataset had a strong degree of taxonomic diversity.

### Performance of ML methods on benchmarking dataset

**Figure 3:**
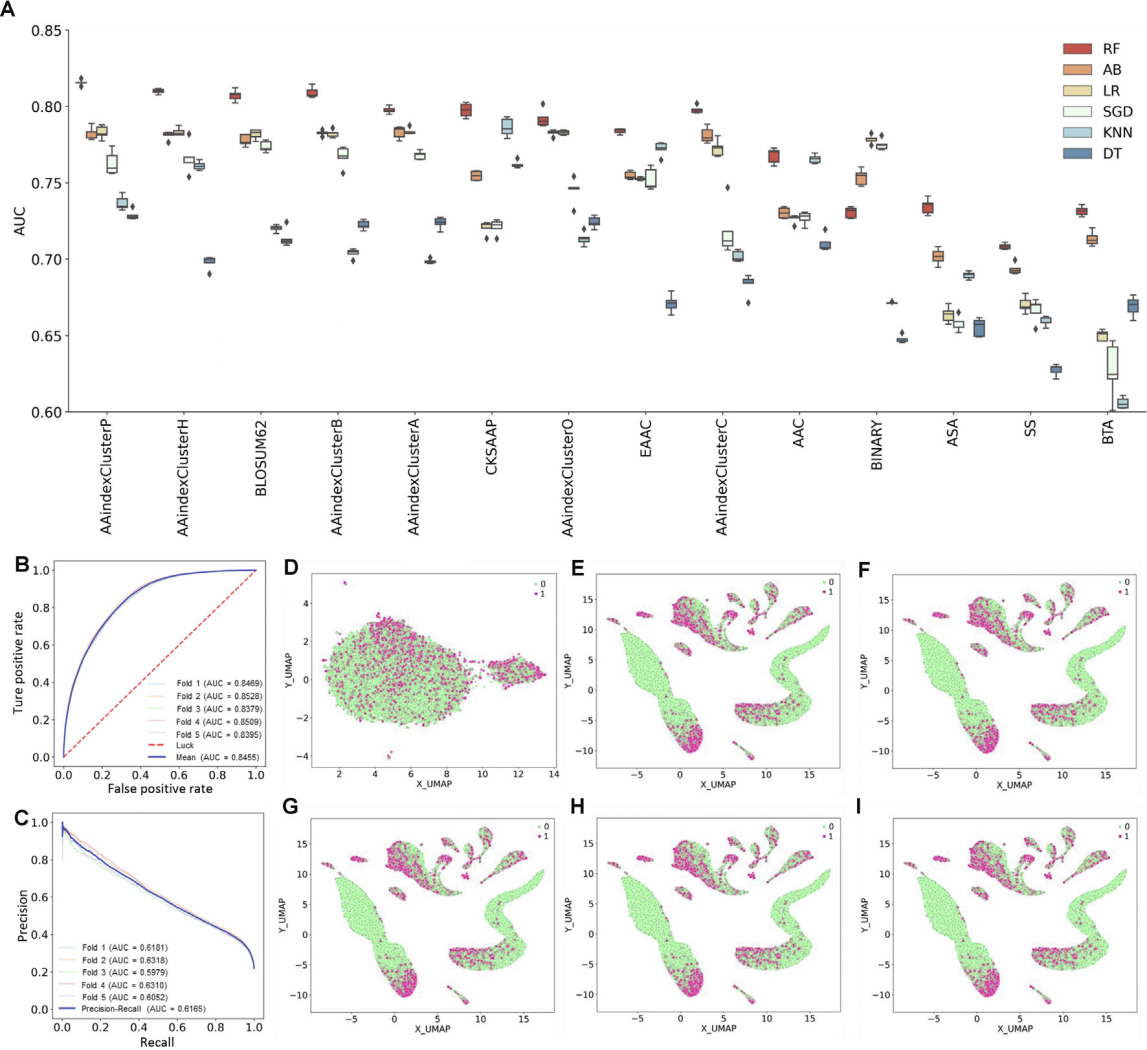
**Performance of NetBCE and otherMLmethods** **A.** Performances of 84 ML models for the 14 types of features. The AUC values were calculated by five-fold CV. **B.** ROC curves for NetBCE by different fold CV. **C.** PR curves for NetBCE by different fold CV. **D.** Feature representation of the epitopes and non-epitopes using the UMAP method in the input layer of NetBCE. **E.** Feature representation of the epitopes and non-epitopes in the CNN layer. **F*.*** Feature representation of the epitopes and non-epitopes in the BLSTM layer. **G.** Feature representation of the epitopes and non-epitopes in the attention layer. **H.** Feature representation of the epitopes and non-epitopes in the fully connected layer. **I.** Feature representation of the epitopes and non-epitopes in the final classification layer. ML, machine learning; CV, cross-validation; AAC, amino acid composition; CKSAAP, composition of K-spaced amino acid pairs; EAAC, enhanced amino acid composition; ASA, accessible surface area; SS, secondary structure; BTA, backbone torsion angle; AB, AdaBoost; DT, decision trees; KNN, k-nearest neighbors; LR, logistic regression; RF, random forest; SGD, stochastic gradient descent; AUC, area under the receiver operating characteristic curve; ROC, receiver operating characteristic; PR, precision–recall; UMAP, Uniform Manifold Approximation and Projection.

So far, numerous tools have been developed for linear BCE prediction. In those tools, a series of sequence or structural features have been adopted. We explored six different conventional ML algorithms, including AB, DT, SGD, KNN, LR, and RF, using 14 different encoding schemes. For each feature, each algorithm was implemented and optimized using five-fold CV on the training dataset. We repeated five-fold CV ten times by randomly portioning the training dataset. The performances of these 84 ML methods in terms of AUC are shown in Figure 3A and Table S5. The average AUC values of five-fold CV of six ML algorithms ranged from 0.695 (DT) to 0.777 (RF). RF, AB, and LR performed better than other ML-based methods (SGD, KNN, and DT), Next, we studied the average performance for each feature among the six ML methods. The AUC values of five-fold CV ranged from 0.666 (BTA) to 0.768 (AAindexClusterP). Thus, different types of features displayed various accuracies for BCE prediction and all the sequence-derived features, physicochemical features, and structural features were informative. We further found that the sequence-derived features performed better compared to structural features. Due to the limitation of protein structure information, three types of structural features were calculated through computational prediction from protein sequences in this study, and thus, the predicted features might lead to a lower prediction accuracy.

### NetBCE for accurate prediction of linear BCEs in proteins

Deep learning has been recently demonstrated to have powerful capability for mining large but complex biomedical data, including image and sequence information extraction and natural language processing. With sufficient B cell assays, the deep neural network can automatically learn informative classification features, making it very appropriate for linear BCE prediction. In this study, a deep learning-based predictor was introduced, called NetBCE, for BCE prediction in the proteins. The NetBCE was implemented with five components: the input layer, convolution–pooling modules, BLSTM layer, attention layer, and the output layer. To evaluate the prediction performance of NetBCE, the five-fold CV was performed on the training dataset. The ROC curves were drawn and the corresponding AUC values were calculated. We found that NetBCE had high performance with the average AUC value of 0.8455 by five-fold CV, with a range from 0.8379 to 0.8528 (Figure 3B). Since the numbers of epitopes and non-epitopes were not balanced in the training dataset, we also performed precision–recall (PR) analysis and calculated the corresponding AUC values. The PR curve indicates the trade-off between the amount of false positive predictions compared to the amount of false negative predictions. NetBCE achieved an average PR AUC value of 0.6165 (Figure 3C), suggesting that our model had great potential in predicting functional epitopes with the high precision.

As above, we drew a conclusion that NetBCE was both faithful and robust for the prediction of linear BCEs, which might be partly attributed to its deep neural network architecture. NetBCE utilized several excellent deep learning modules, *e.g.*, CNN, BLSTM, and attention, to learn a more efficient and interpretive representation of the epitope sequence hierarchically. To elucidate the capability of hierarchical representation by NetBCE, we visualized the epitopes and non-epitopes using UMAP method based on the feature representation at varied network layers. We found that the feature representation displayed more discriminative along the network layer hierarchy (Figure 3D–I). More specifically, the feature representations for epitope and non-epitope sites were mixed at the input layer. As the model continued to train, epitopes and non-epitopes tend to occur in very distinct regions with efficient feature representation.

### Performance evaluation and comparison

**Figure 4:**
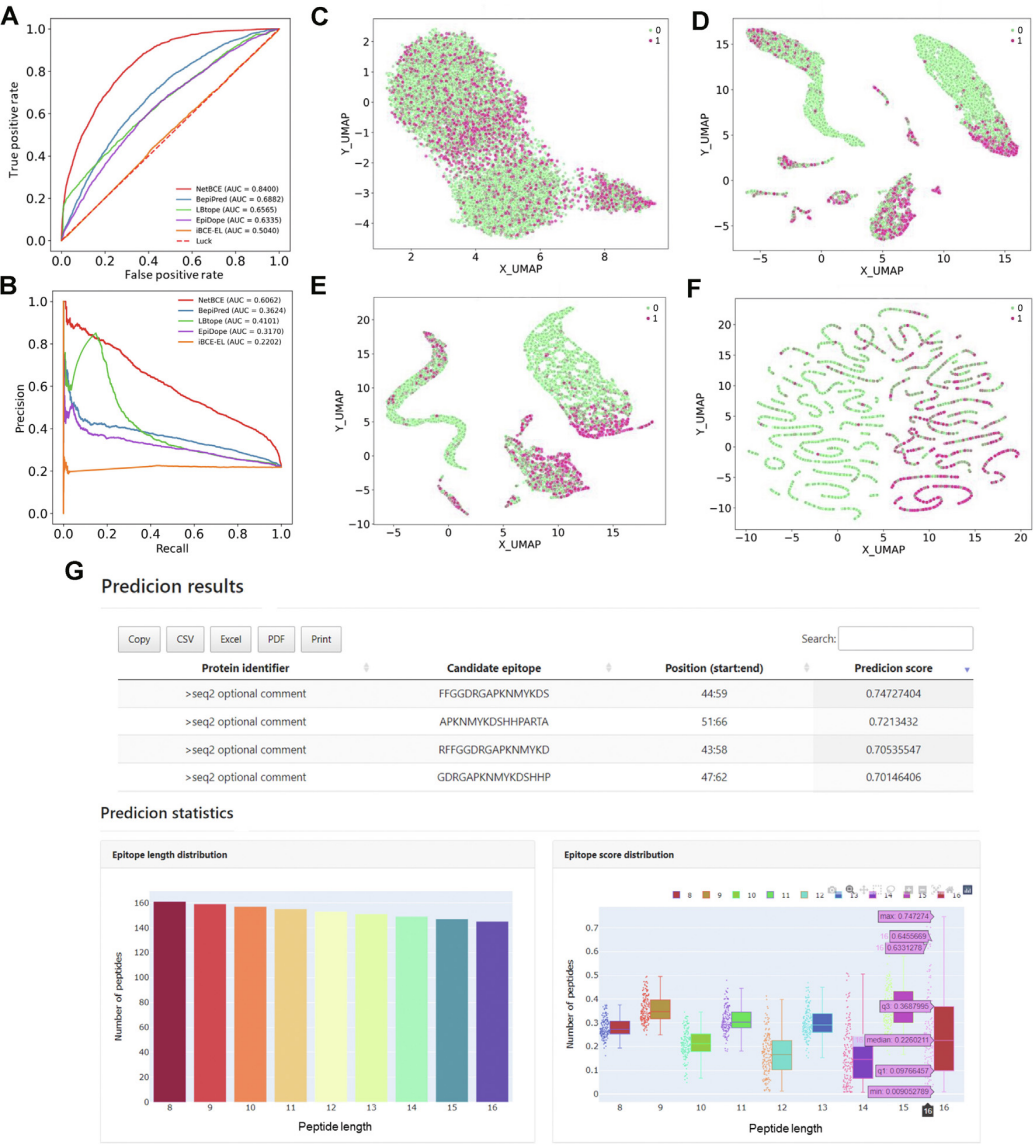
**Performance comparison between NetBCE and other toolsand the display interface of NetBCE software** **A.** Comparison of NetBCE with other predictors, including BepiPred, LBtope, iBCE-EL, and EpiDope on the independent dataset regarding the ROC curves. **B.** Comparison of NetBCE with other predictors regarding the PR curves. **C.** Feature representation of the epitopes and non-epitopes in the independent dataset using the UMAP method in the input layer of NetBCE. **D.** Feature representation of the epitopes and non-epitopes (independent dataset) in the BLSTM layer. **E.** Feature representation of the epitopes and non-epitopes (independent dataset) in the fully connected layer. **F.** Feature representation of the epitopes and non-epitopes (independent dataset) in the final classification layer. **G.** The display interface of NetBCE software. NetBCE provides and visualizes the prediction results in an interactive HTML file using the Python, PHP, JavaScript, and Bootstrap package in an easily readable and interpretable manner.

To demonstrate the superiority of NetBCE, we first compared the performance of NetBCE with other six ML-based methods (AB, DT, SGD, KNN, LR, and RF) by AUC value measure. We observed that the performance of NetBCE was generally better than other six ML-based methods, resulting in the AUC value improvements from 8.77% to 21.58%. We further compared NetBCE to four previously developed and available linear BCE predictors, including LBtope, iBCE-EL, BepiPred, and EpiDope. Since these four tools did not offer the function for customizing prediction models on other B cell assays, the curated independent dataset was straightly entered to each service to calculate the performance and compare with the prediction result by NetBCE. NetBCE had high performance with the AUC value of 0.8400 on the independent dataset (Figure 4A). For BepiPred [15], LBtope [23], iBCE-EL [32], and EpiDope [31] that provide prediction scores for all input, we drew the ROC curves and corresponding AUC values were calculated as 0.6882, 0.6565, 0.5040, and 0.6335, respectively. When compared with the second-best tool BepiPred [15], NetBCE reached an 22.06% AUC value improvement (Figure 4A). Moreover, NetBCE reached PR AUC of 0.6062 on the independent dataset (Figure 4B), which was superior to other existing tools. To elucidate the underlying mechanism of NetBCE leading to superior performance in the independent dataset, we applied NetBCE to predict the output of important network modules in the model and used UMAP to visualize the predicted feature representation at varied network layers (Figure 4C–F). We found that predicted features became more and more distinguishable with the training of the model. Epitopes and non-epitopes in the independent dataset were mixed at the input layer, culminating with a clear separation in the output layer. In comparison, NetBCE implemented by the interpretable deep learning architecture considerably outperformed other existing tools.

### Case study and usage of NetBCE

Considering the severe and still ongoing SARS-CoV-2 pandemic, screening of immunogenic targets against the viral protein is urgently needed for the development of sensitive diagnostic tools and vaccination strategies. Recent studies have well-characterized immunogenic T/B-cell epitopes of SARS-CoV-2 spike protein using linear peptides [52]. In addition to spike protein, open reading frame 8 (ORF8) is a unique protein expressed in SARS-CoV-2 that is also highly immunogenic as reported in COVID-19 patients at both early and late stages of disease [53]. So far, the BCEs of the ORF8 protein remain largely unknown. Here, we used NetBCE to predict candidate BCEs on the ORF8 protein. The sequence that was used for the identification of linear peptides of the ORF8 protein of SARS-CoV-2 was obtained under NCBI Reference Sequence: YP_009724396.1. We set NetBCE to segment and scan a large peptide library consisting of 15-mer peptides overlapping by 14 amino acids spanning the ORF8 sequence. As a result, a total of 107 epitopes were screened (Table S6). These predicted linear BCEs may provide some insights into the design of serological diagnostics and peptide-based vaccination approach for fighting this COVID-19 pandemic.

In addition, we developed a tool to provide function for linear BCE prediction based on the NetBCE model. The NetBCE tool is available at https://github.com/bsml320/NetBCE. NetBCE provides and visualizes the prediction results in an interactive HTML file using the Python, PHP, JavaScript, and Bootstrap package in an easily readable and interpretable manner. Users can input the candidate proteins in a FASTA format. In addition, users need to select one or more peptide lengths so that NetBCE can construct a library of candidate epitope peptides. For an example of the output page in Figure 4G, NetBCE provides a probability score for each candidate peptide with its value in a range from 0 to 1. All prediction results can be copied, printed, and downloaded in three formats: “CVS”, “Excel”, and “PDF”. NetBCE also provides another two interactive HTML plots to show the distribution of lengths and scores for all candidate peptides.

## Discussion

In this study, we first compiled an experimentally well-characterized dataset, containing more than 124,000 experimentally linear epitope-containing regions from 3567 protein clusters, through a widely used immunization database (IEDB). Based on the curated benchmark dataset, 14 features were encoded including five sequence-based features, six physicochemical property-based features, and three structural features. All features were evaluated by six conventional ML algorithms, and the AUC values were calculated through five-fold CV. Our result revealed that predictive power for linear BCE prediction varied greatly by different types of features, and all the sequence-derived features, physicochemical features, and structural features were informative. It should be noted that when the structural information is very limited and obtained by prediction in this study, we found that sequence-derived features and physicochemical features performed better, but structural features were also very important for functional epitope prediction. This is because over 80% known BCE residues recognized by antibodies are structural/conformational rather than sequential. Building on this large data collection, a ten-layer deep learning framework, named NetBCE, was implemented. NetBCE was built by five components: the input layer, convolution–pooling modules, BLSTM layer, attention layer, and the output layer. To assess the performance of NetBCE, we performed the five-fold CV on the training dataset. NetBCE had high performance with the average AUC value of 0.8455, with a range from 0.8379 to 0.8528, by automatically learn informative classification features. In comparison, NetBCE outperformed conventional ML methods by increasing the AUC value by a range of 8.77%–21.58% in the same training dataset. Moreover, NetBCE had high performance with the AUC value of 0.8400 on the independent dataset, and achieved over 22.06% improvement of AUC value for the linear BCE prediction compared to other tools. Compared to the black box of training process in traditional ML, the interpretability of our model is also easier to explore. To elucidate the capability of hierarchical representation by NetBCE, we visualized the epitopes and non-epitopes based on the predicted feature representation at varied network layers. We found the feature representation came to be more discriminative along the network layer hierarchy, demonstrating that our model has excellent classification ability.

In the future, we will continuously strengthen NetBCE by collecting more experimentally identified BCEs into the training dataset. Although the dataset included in the current database is getting larger, a considerable number of BCEs might be false positives that do not have sufficient positive test results. The development of methods for data quality control currently remains a great challenge to minimize the false positives caused in various types of experimental assays. We indeed found a number of epitope-containing regions with non-uniform test results that had both positive and negative responses when we processed the data. Thus, to build a high-quality dataset of epitopes and non-epitopes, a strict criterion was adopted in this study, like what was applied in BepiPred2 tool. Specifically, we first obtained all test records for each epitope-containing region. We defined all epitope-containing regions that were tested positive by at least two assays as epitopes to avoid possible chance of false positive from a single assay. Moreover, all epitope-containing regions that tested in at least two assays and were not tested as positive in any assay were considered as non-epitopes. All other epitope-containing regions with inconsistent test responses that did not meet both criteria were excluded. This strategy can not only retain as many high-quality epitopes as possible, but also eliminate as much as possible the epitope-containing regions with contradictory test responses.

Usually, a high epitope probability outputted by NetBCE does not mean a strong immunity. Because NetBCE is a classification model, its training data are labeled as “yes” or “no”. Therefore, to link the predicted probability and immunity, we need to build a regression model. By doing so, it needs a training set with measurements of binding affinity as the label, but this part of the data is not currently available. However, regarding this potential application, we still have a way to screen more immunogenic BCEs using NetBCE. It has been noted that the BCEs with nearby CD4^+^ T-cell epitopes are more likely to be truly immunogenic and to induce mature BCRs and antibodies, a phenomenon known as T–B reciprocity [54]. With this biological dependency, we can predict both candidate BCEs and nearby CD4^+^ T-cell epitopes (*e.g.*, using netMHCIIpan software [55]), and combinations with high scores for both have higher chances of being immunogenic.

Moreover, more useful features and advanced deep neural network frameworks will be adopted for the development of model for linear BCEs. For example, post-translational modifications (PTMs), including glycosylation, phosphorylation, and acetylation, can alter protein structure and further affect the recognition of between epitopes and antibodies [56]. Integrating PTM information can help improve the prediction of functional epitopes. To do so, we may need to download and integrate the experimentally validated PTM sites from public databases, such as dbPTM [57], PhosphoSitePlus [58], Eukaryotic Phosphorylation Sites Database (EPSD) [59], and Protein Lysine Modifications Database (PLMD) [60]. Then, BCEs and flanking sequences can be scanned to search the known PTM sites. By counting the number of PTM sites that are 10–20 positions away from the BCE boundaries and PTM sites within the BCEs, we can construct multiple numerical features for different PTM types. We thus can combine these PTM features and representations obtained by deep learning to further improve the prediction of functional BCEs. Moreover, we can obtain PTM-related amino acid indexes from AAindex database and integrate these features to construct a more comprehensive model. Taken together, this study reported a novel and accurate approach for the prediction of linear BCEs. We anticipate that the interpretable deep neural network can be easily extended to other sequence-derived prediction task to corroborate much better prediction.

## Code availability

The source codes are implemented in Python and are freely available at GitHub (https://github.com/bsml320/NetBCE) and BioCode (https://ngdc.cncb.ac.cn/biocode/tools/BT007321).

## CRediT author statement

**Haodong Xu:** Conceptualization, Methodology, Software, Data curation, Visualization, Writing - original draft. **Zhongming Zhao:** Conceptualization, Methodology, Project administration, Supervision, Writing - review & editing, Funding acquisition. Both authors have read and approved the final manuscript.

## Competing interests

The authors have declared no competing interests.

## Supplementary material

Supplementary Table S1 **Summary of 25 existing methods for the prediction of linear B-cell epitope**

Supplementary Table S2 **The training dataset of experimentally-verified epitope-containing regions for NetBCE and other 84 constructed machine learning models**

Supplementary Table S3 **The testing dataset of experimentally-verified epitope-containing regions for performance comparison**

Supplementary Table S4 **Summary of the 14 types of features used in this study**

Supplementary Table S5 **The five-fold cross-validation results of 84 machine learning models using the 14 types of features for linear B-cell epitope prediction**

Supplementary Table S6 **Prediction of candidate B-cell epitopes on open reading frame 8 protein of SARS-CoV-2 by NetBCE**
